# Chondrogenic Commitment of Human Bone Marrow Mesenchymal Stem Cells in a Perfused Collagen Hydrogel Functionalized with hTGF-β1-Releasing PLGA Microcarrier

**DOI:** 10.3390/pharmaceutics13030399

**Published:** 2021-03-17

**Authors:** Erwin Pavel Lamparelli, Joseph Lovecchio, Maria Camilla Ciardulli, Valentina Giudice, Tina P. Dale, Carmine Selleri, Nicholas Forsyth, Emanuele Giordano, Nicola Maffulli, Giovanna Della Porta

**Affiliations:** 1 Department of Medicine, Surgery and Dentistry, University of Salerno, via S. Allende, 84081 Baronissi, SA, Italy; elamparelli@unisa.it (E.P.L.); mciardulli@unisa.it (M.C.C.); vgiudice@unisa.it (V.G.); cselleri@unisa.it (C.S.); nmaffulli@unisa.it (N.M.); 2 Department of Electrical, Electronic and Information Engineering “Guglielmo Marconi” (DEI), University of Bologna, via dell’Università 50, 47522 Cesena, FC, Italy; joseph.lovecchio@unibo.it (J.L.); emanuele.giordano@unibo.it (E.G.); 3 Health Sciences and Technologies-Interdepartmental Center for Industrial Research (HST-ICIR), University of Bologna, via Tolara di Sopra 41/E, 40064 Ozzano dell’Emilia, BO, Italy; 4 Guy Hilton Research Centre, School of Pharmacy and Bioengineering, Keele University, Stoke-on-Trent, Staffordshire ST4 7QB, UK; t.p.dale@keele.ac.uk (T.P.D.); n.r.forsyth@keele.ac.uk (N.F.); 5 Advanced Research Center on Electronic Systems (ARCES), University of Bologna, via Vincenzo Toffano 2/2, 40125 Bologna, BO, Italy; 6 Research Centre for Biomaterials BIONAM, Università di Salerno, via Giovanni Paolo II, 84084 Fisciano, SA, Italy

**Keywords:** human bone marrow mesenchymal stem cells, hTGF-β1 controlled delivery, PLGA microcarriers, 3D collagen scaffold, chondrogenic commitment, perfusion bioreactor system

## Abstract

Tissue engineering strategies can be relevant for cartilage repair and regeneration. A collagen matrix was functionalized with the addition of poly-lactic-co-glycolic acid microcarriers (PLGA-MCs) carrying a human Transforming Growth Factor β1 (hTFG-β1) payload, to provide a 3D biomimetic environment with the capacity to direct stem cell commitment towards a chondrogenic phenotype. PLGA-MCs (mean size 3 ± 0.9 μm) were prepared via supercritical emulsion extraction technology and tailored to sustain delivery of payload into the collagen hydrogel for 21 days. PLGA-MCs were coseeded with human Bone Marrow Mesenchymal Stem Cells (hBM-MSCs) in the collagen matrix. Chondrogenic induction was suggested when dynamic perfusion was applied as indicated by transcriptional upregulation of COL2A1 gene (5-fold; *p* < 0.01) and downregulation of COL1A1 (0.07-fold; *p* < 0.05) and COL3A1 (0.11-fold; *p* < 0.05) genes, at day 16, as monitored by qRT-PCR. Histological and quantitative-immunofluorescence (qIF) analysis confirmed cell activity by remodeling the synthetic extracellular matrix when cultured in perfused conditions. Static constructs lacked evidence of chondrogenic specific gene overexpression, which was probably due to a reduced mass exchange, as determined by 3D system Finite Element Modelling (FEM) analysis. Proinflammatory (IL-6, TNF, IL-12A, IL-1β) and anti-inflammatory (IL-10, TGF-β1) cytokine gene expression by hBM-MSC was observed only in dynamic culture (TNF and IL-1β 10-fold, *p* < 0.001; TGF-β1 4-fold, *p* < 0.01 at Day 16) confirming the cells’ immunomodulatory activity mainly in relation to their commitment and not due to the synthetic environment. This study supports the use of 3D hydrogel scaffolds, equipped for growth factor controlled delivery, as tissue engineered models for the study of in vitro chondrogenic differentiation and opens clinical perspectives for injectable collagen-based advanced therapy systems.

## 1. Introduction

Articular cartilage injuries are a significant health problem due to their poor reparative potential. Osteoarthritis (OA) is caused by progressive joints wear, and is often a consequence of these injuries [1]. The incidence of OA increases with age and it is now one of the most prevalent diseases in elderly people [2,3]. The avascular nature of cartilage prevents spontaneous healing, increasing the potential for chronic damage, where conventional pharmacological therapy is directed toward symptomatic treatment and pain reduction, and not to regeneration of impaired cartilage [4]. Patients who do not respond to pharmacological intervention ultimately require surgical procedures to promote cartilage repair, such as arthroscopic debridement, bone marrow (BM) stimulation techniques, autologous chondrocyte implantation, and, finally, total or partial joint replacement [5]. BM stimulation requires subchondral level drilling to initiate bleeding, which enables mesenchymal cells from the bone marrow to migrate into the site of injury. However, subsequent tissue repair is primarily composed of fibrocartilage, with accompanying poorer biochemical and biomechanical characteristics than hyaline cartilage. Consequently, the clinical outcomes are not always adequate [6].

Tissue engineering (TE) provides an alternative strategy to traditional therapeutic approaches and offers potential to improve joint cartilage therapy [7]. TE describes the application of biocompatible and biodegradable polymers as 3D scaffolds to support cell growth and differentiation. The scaffold has become a focus of interest where tailoring mechanical or biochemical properties to replicate the native natural extracellular matrix (ECM) is becoming a key goal. The ECM is itself a dynamic network providing mechanical force to support tissue function and the reproduction of its physical and biochemical properties represents a major TE challenge [8]. The ECM of articular cartilage is composed mainly of hydrated collagen (i.e., type II) and proteoglycans (i.e., aggrecan), with a high water content to assure and maintain its specific mechanical properties [9].

TE scaffolds are generally composed of biodegradable and biocompatible materials, which may be natural or synthetic, where the correct choice of scaffold is crucial to the success of the therapeutic strategy [10]. Collagen-based scaffolds are appropriate to host chondrocytes due to their similarity to native ECM. However, hydrogels exhibit low mechanical resistance and consequently are often cross-linked to create adequate stiffness [11,12]. Particularly, type I collagen hydrogels are frequently investigated as being suitable for implantation given their in vivo biocompatibility [13]. Furthermore, cells in a 3D culture environment are subjected to lower oxygen content; indeed, they receive fewer exchanges from the external medium and tend to be in a hypoxic state [14]. Hypoxia has been also reported to be favorable for the human mesenchymal stem cell (hMSC) induction towards both chondrogenic and tenogenic phenotypes [15,16].

On the other hand, the induction of chondrogenic differentiation frequently depends on the supplementation of growth factors such as TGF-β1 and TGF-β3. Of these, TGF-β1 is the most widely investigated for the promotion of chondrogenesis in hMSCs and human adipose stem cells (hASCs); however, its dose–response effect remains still unclear. In relation to this, hBM-MSCs cultured with a TGF-β1 concentration ranging from 0.01 to 0.1 ng/mL reveals no evidence of chondrogenic differentiation [17,18]. In contrast, hASCs are induced by 10 ng/mL TGF-β1 supplementation into aggregates with the expression of sulfated proteoglycans and aggrecan, while 100 ng/mL reduces this chondrogenic phenotype [19]. Growth factors are routinely administered via a supplemented medium which has to be replaced frequently, with a high economic impact due to the relevant cost of the recombinant peptides.

Recent attention has focused on the encapsulation of growth factors into biopolymeric micro/nanocarriers to promote their controlled local release within 3D microenvironments for TE application [20,21,22]. Micro/nanocarriers’ fabrication can be based on emulsion processing by the evaporation or extraction of oily phase and the subsequent collection of the resulting biopolymer beads. Dense gas technology has also been described for oily phase extraction, allowing accurate carrier size control and loading of several active substances [23,24,25]. Carriers can be immobilized with ease within a hydrogel matrix, and controlled delivery of their cargo within the 3D scaffold accomplished. This creates the possibility of the optimized control of the constant growth factor delivery within a 3D system [26].

Dynamic conditions are an essential component of 3D culture systems [20,27,28]. Perfusion upregulates chondrogenic markers [29,30], preserves cartilage phenotype, and preventes the hypertrophy of MSC-derived chondrocytes, which remains a fundamental problem in cell-based strategies [31]. Furthermore, when micro- and nanocarriers are used for controlled delivery of growth factors, an adequate release is promoted by the dynamic environment, whereas in static conditions, reduced mass transfer can decrease the extent of drug release, impacting scaffold efficacy in driving cell commitment [32].

Following these considerations, the present study aimed to evaluate the potential of microcarrier functionalized 3D collagen scaffolds, as a bioengineering tool, for the induction and maintenance of the chondrogenic commitment of human stem cells. The attention will focus on the fabrication of PLGA carriers for hTGFβ1 controlled release, the assembly of 3D scaffold systems with human Bone Marrow Mesenchymal Stem Cells (hBM-MSC) and their 3D culture along 16 days in both static and dynamic conditions. Gene expression data by qRT-PCR and histological and quantitative-immunofluorescence (qIF) analysis assays will provide indications on cells’ behavior at given time points, whereas cytokines’ gene expression can suggest cells reaction to the 3D environment.

## 2. Materials and Methods

### 2.1. hBM-MSCs Isolation and Harvesting

Briefly, total BM aspirate was directly seeded at a concentration of 50,000 total nucleated cells/cm^2^ in T75 plastic flask in Minimum Essential Medium Alpha (α-MEM) supplemented with 1% Glutagro^TM^, 10% Fetal Bovine Serum (FBS) and 1% Penicillin/Streptomycin (Pen/Strep) and incubated at 37 °C in an atmosphere of 5% CO_2_ and 95% relative humidity [33]. After 72 h, nonadherent cells were removed by medium change and the remaining adherent cells were then fed twice weekly thereafter with new medium. On day 14, colonies of adherent hBM-MSCs were detached and reseeded at 4000 cells/cm^2^ in the same culture conditions. Once cell cultures reached 70–80% confluence (7–8 days from the previous passage), cells were detached using 0.05% trypsin-0.53 mM Ethylenediaminetetraacetic acid (EDTA) and washed with 1× of phosphate buffered saline (PBS) (Corning Cellgro, Manassas, VA, USA), counted using Trypan Blue (Sigma-Aldrich, Milan, Italy) and subcultured at a concentration of 4 × 10^3^ cells/cm^2^. At passage 2, cells were used experimentally. 

Flow cytometry analysis was performed on *h*BM-MSCs obtained at passage 2; cells were positive for CD90, CD105, CD73 and negative for CD14, CD34, CD45, HLA-DR expression (Beckman Coulter, Brea, CA, USA) (data not shown) [34].

### 2.2. PLGA-MCs Fabrication by Supercritical Emulsion Extraction Technology

PLGA-MCs were obtained using Supercritical Emulsion Extraction (SEE) technology via dense gas extraction of emulsion oily phase. The process layout is described by a countercurrent packed tower operating in continuous mode [35]. PLGA-MCs were fabricated using a water–oil–water emulsion (ratio 0.25:5:50). 

In detail, recombinant hTGF-β1 (PeproTech EC, Ltd., London, UK) was dissolved into 0.1% *w*/*v* human serum albumin (hSA; Sigma-Aldrich, Milan, Italy) containing 0.06% *w*/*w* of polyvinyl alcohol (PVA; Sigma-Aldrich, Milan, Italy) as surfactant, hSA was included as growth factor stabilizer in the primary emulsion [36]. The water phase was added to the oily phase formed in ethyl acetate (EA, purity 99.9%) and PLGA (50:50; RESOMER^®^ RG 504H, 0.45–0.60 [dL/g] from EVONIK Nutrition and Care GmbH, Darmstadt, Germany) at 5% *w*/*w*. The water/oil (*w*/*o*) emulsification was produced by vortex mixer for 30 s at maximum speed with the resulting primary emulsion immediately poured into an EA-saturated aqueous solution containing 0.1% *w*/*w* Tween 80 and 15% *w*/*w* glucose. The secondary emulsion was then formed via a high-speed homogenizer (mod. L4RT; Silverson Machines Ltd., Waterside, Chesham Bucks, UK) at 2000 rpm for 5 min and at 10 °C. All emulsions were processed by SEE immediately after their preparation. 

Operative pressure and temperature conditions in the high-pressure column were set at 8 MPa and 38 °C, respectively, with a dense gas flow of Carbon Dioxide (CO_2_) set at 1.4 kg/h with Liquid/Gas ratio of 0.1 *w*/*w* [37]. PLGA-MC suspensions were collected at the bottom of the extraction column, washed to eliminate surfactant, and lyophilized. All washing steps were performed in sterile conditions supplemented with a pen/strep and amphotericin B (1% *w*/*v*) solution.

### 2.3. Carrier Size Distribution and Morphological Analyses

Particle size distributions (PSDs) of PLGA-MC suspensions were measured using laser granulometer (mod. Mastersizer S; Malvern Instruments Ltd., Worcestershire, UK), based on dynamic light scattering (DLS). Sizes are expressed as volume mean (MS) with standard deviation (SD) in nanometers (nm). The shape and morphology of the PLGA-MCs were investigated by field emission-scanning electron microscopy (FE-SEM, mod. LEO 1525; Carl Zeiss SMT AG, Oberkochen, Germany). PLGA-MC samples were placed on double-sided adhesive carbon tape previously glued to an aluminum stub and coated with a gold film (250 A thickness) using a sputter coater (mod.108 A; Agar Scientific, Stansted, UK).

### 2.4. hTGF-β1 Release Study

The release profile of hTGF-β1 from PLGA-MCs was monitored in vitro for 21 days from 5 ± 0.3 mg of PLGA-MCs suspended in 500 µL of PBS 1× containing 0.1% Tween 20 and 0.1% hSA, placed in an incubator at 37 °C, and stirred continuously at 100 rpm. Every 24 h, the samples were centrifuged at 14,000 rpm for 30 min, and the supernatant completely removed and replaced with fresh PBS to maintain sink conditions. Released hTGF-β1 concentrations from collected samples were then measured with an Enzyme Linked Immunosorbent Assay (ELISA cat. no. SEA124Hu; Cloud-Clone Corp., Katy, TX, USA). Release experiments were performed in triplicate (n = 3), and the curve describing the mean profile calculated as ng/g (protein released/PLGA-MCs) versus time or as a percentage amount calculated with respect to the maximum growth factor load.

### 2.5. 3D Collagen Scaffolds Preparation and Characterization

For each scaffold, a mixture of type I collagen derived from rat tail (cat. no 5153, Advanced BioMatrix, Carlsbad, CA, USA), neutralization solution (cat. no 5155, Advanced BioMatrix, Carlsbad, CA, USA), and α-MEM (Corning, NY, USA) was added at a 9:1:2 ratio. This was enriched via addition of 22 mg/mL of PLGA-MCs (7 ug/g hTGF-β1/PLGA) and 1 × 10^6^ cells/mL creating a scaffold mastermix. A 300 µL volume of mastermix was pipetted drop by drop into cylindrical molds (5 × 5 mm diameter vs. height) then placed into an incubator at 37 °C for 60 min to allow collagen thermal cross-linking. The obtained 3D bioengineered scaffolds (0.3% *w*/*w* collagen) were then transferred into culture plates.

Cell viability in scaffolds was detected by fluorescence live/dead assay (Calcein AM solution (Cat. no C1359) and Ethidium homodimer I solution (Cat. no E1903), Sigma-Aldrich, Milan, Italy), immediately after making scaffold. Cells were stained for 1 h at 37 °C, washed in PBS 1× and imaged in a fluorescence microscope (mod. Eclipse, Nikon Corporation, Tokyo, Japan).

The internal morphology of the scaffold was observed by field emission-scanning electron microscopy (FE-SEM) (mod. LEO 1525; Carl Zeiss SMT OG, Oberkochen, Germany). Samples were first fixed in 4% (*w*/*v*) paraformaldehyde (PFA) (4 °C, overnight), and then dehydrated by multiple passages across ethanol: water solutions (10 min each) with increasing percentages of ethanol (10%, 20%, 30%, 50%, 70%, 90%), ending in a 100% dehydrating liquid (3 changes, 10 min each), and lyophilized using a critical point dryer (mod. K850 Emitech, Assing, Rome, Italy). Samples were first immersed in liquid nitrogen until freezing, and then fractured with a needle in order to expose their internal surface, placed on a double-sided adhesive carbon tape previously glued to an aluminum stub, and coated with a gold film (250 A thickness) using a sputter coater (mod.108 A; Agar Scientific, Stansted, UK), before observation.

#### 2.5.1. Static and Dynamic Culture

For static condition, 3D scaffolds were placed into a multiwell plate, whereas for the dynamic stimulation, a custom perfusion bioreactor was used. The bioreactor was formed by a multiwell plate milled in poly(methyl methacrylate) (Altuglas^®^ CN, La garenne-colombes France), a biocompatible material for biomedical applications [38]. The plate featured two holes allowing the insertion of silicon tubes (Tygon^®^, Charny, France) for flow provision via peristaltic pumps at a constant flow rate of 1.0 mL/min [39]. This continuous flow was maintained during dynamic experiment. The chondrogenic medium was recycled from the peristaltic pump and changed twice a week with fresh medium. The bioreactor system operated within a standard cell culture incubator.

#### 2.5.2. Hematoxylin and Eosin and Sirius Red Staining

Scaffold sections (15 μm thickness) were hydrated using a decreasing ethanol gradient to 75% and then washed for 5 min in water. Hematoxylin and eosin (H&E) staining and Picrosirius Red Stain Kit (Polysciences, Inc., Warrington, PA, USA) were used for 60 min. Samples were dehydrated using an increasing ethanol gradient and cleared in xylene for 5 min. Finally, sections were mounted using Eukitt (Sigma-Aldrich, Milan, Italy) mounting medium. Picrosirius red brightfield and polarized light images were acquired with a Brunel polarization microscope equipped with a Nikon D500 camera.

#### 2.5.3. Immunofluorescence Assay

Collagen scaffold was fixed in 4% paraformaldehyde (PFA) for 2 h at room temperature, cryo-protected in 30% sucrose (4 °C, overnight), included in optimal cutting temperature (OCT) compound, and cut in slices of 15 μm of thickness using a cryostat (mod. CM1950, Leica, Wetzlar, Germany). Slices were permeabilized with 0.1% Triton X-100 for 10 min and blocked with Bovine Serum albumin (BSA) solution (1% *w*/*v*) for 1h. For type II and type III collagen staining, slices were incubated overnight at 4 °C with a rabbit polyclonal anticollagen type II antibody (1:100, Abcam, Cambridge, UK) and mouse polyclonal anticollagen type III antibody (1:100; Santa Cruz Biotech., Santa Cruz, CA, USA). Following incubation with the primary antibody, slices were incubated for 1h at RT with the Alexa Fluor ^TM^ 488 goat antirabbit IgG (1:400, Thermo Fisher Sci., Waltham, MA, USA) and the DyLight 649 antimouse IgG (1:500, BioLegend, San Diego, CA, USA) antibodies. Cell nuclei were counterstained with 4′,6-diamodino-2-phenylindole (DAPI). For type I collagen staining, slices were incubated overnight at 4 °C with a rabbit polyclonal anticollagen type I antibody (1:100, Abcam, Cambridge, UK); then, Alexa Fluor ^TM^ 488 goat antirabbit IgG (1:400, Thermo Fisher Sci., Waltham, MA, USA) antibody was used.

Single images were acquired with identical light intensity, exposure time and gain settings, using an inverted Leica laser-scanning confocal microscope (mod. TCS SP5; Leica Microsystems, Wetzlar, Germany) equipped with a plan Apo 63X/1.4 NA oil immersion objective. Signals intensity was quantified using the software ImageJ (rel.1.52p National Institutes of Health, Bethesda, MD, USA). Original images in RGB format were converted into a 16-bit (gray scale) format. Thereafter, the tagged areas were expressed as an average value of pixel intensity within a range from 0 (dark) to 255 (white). Data were normalized to the number of cells present in the whole field [40]. Ten images of several fields were used for the analysis at each time point. All data were reported as fold change relative to untreated cells.

### 2.6. RNA Isolation and Gene Expression Profile

Total RNA was extracted from *h*BM-MSCs seeded into both 12 well-plates and collagen scaffolds using QIAzol^®^ Lysis Reagent (Qiagen, Hilden, Germany), chloroform (Sigma-Aldrich, Milan, Italy) and the RNeasy Mini Kit (Qiagen, Hilden, Germany). For each sample, 1 μg of total RNA was reverse transcribed using the iScriptTM cDNA synthesis kit (Bio-Rad, Milan, Italy). 

Relative gene expression analysis was performed in a LightCycler^®^ 480 Instrument (Roche, Italy), using the SsoAdvanced^TM^ Universal SYBR^®^ Green Supermix (Bio-Rad, Foster City, CA, USA) with the validated primers for COL1A1, COL2A1, COL3A1, SOX9, ACAN (Bio-Rad, Foster City, CA, USA) and for proinflammatory cytokines IL-6, TNF, IL-12A, IL-1β and anti-inflammatory ones IL-10, TGF-β1 (Bio-Rad, USA) following MIQE guidelines [41]. Amplification was performed in a 10 μL final volume, including 2 ng of complementary DNA (cDNA) as template. Specificity of the formed products was addressed via melting curve analysis. Triplicate experiments were performed for each condition explored, and data were normalized to glyceraldehyde-3-phosphate dehydrogenase (GAPDH) expression (reference gene), applying the geNorm method [42] to calculate reference gene stability between the different conditions (calculated with CFX Manager software; M < 0.5). Fold changes in gene expression were determined by the 2^−ΔΔCP^ method and are presented as relative levels versus untreated cells at each explored time-point.

### 2.7. FEM Modeling

Finite Element Modeling (FEM) was implemented using COMSOL Multiphysics Software to assess nutrient consumption and waste production during the cell culture. Mass transport based on the second Fick law was modeled for both static and dynamic regimes, where all components were obtained using primitive geometries and Boolean operations that considered both well and scaffold to have a cylindrical geometry (well = 22 mm in diameter and 7.4 mm in height; scaffold 5 mm in diameter and 5 mm in height). Assumed boundary conditions were laminar flow and mass transport with a permeability coefficient of hydrogel assumed as 2 × 10^−9^ cm^2^ [43]. 

Other mass transport parameters are reported in Table 1. At steady-state conditions, the concentrations of nutrient (consumed) and waste (produced) variation were simulated taking into account a flow rate of 1 mL/min and considering the different diffusion parameters across the medium or across the hydrogel. A sensitivity study of the mesh addressed the most computationally efficient solution. The boundary conditions, constants and main parameters used in the model were derived from previous FEM simulation data [39].

### 2.8. Statistical Analysis

Results, obtained from multiple experiments (n = 3 biological replicates), are presented as mean ± standard deviation (SD). Statistical analysis was performed using the two-tailed independent Student’s T test for comparisons of two independent groups. To compare the influences of the chemical and dynamic stimulation within the same time point, two-way ANOVA was used followed by Tukey’s multiple comparison test. *p* values less than 0.05 were accepted as significant [44]. All statistical analysis was conducted using GraphPad Prism software (6.0 for Windows).

## 3. Result and Discussion

### 3.1. PLGA Carriers Characterization and hTGF-β1 Release Profile

**Figure 1:**
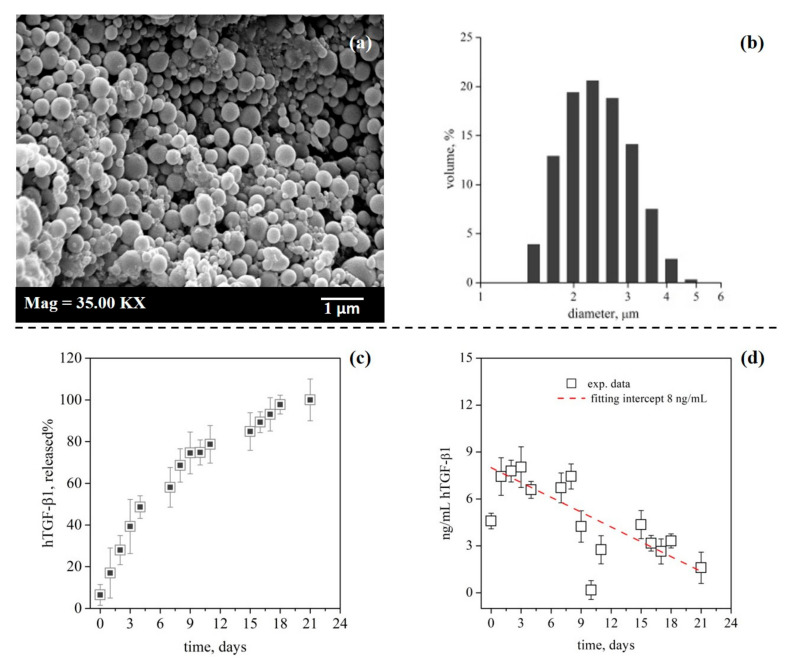
Characterization of carriers: morphology, particle size distribution and hTGF-β1 release profiles for PLGA-MCs. SEM image (**a**) and particle size distribution (**b**) of PLGA carriers obtained after SEE process; size distribution data are expressed as volume percentage. hTGF-β1 release profiles expressed as percentage of total load and monitored at 37 °C and 100 rpm by ELISA-based assay; n = 3 (**c**); hTGF-β1 amount (ng/mL/day) released within each 3D-scaffold loaded with 6.6 mg of carriers in dynamic condition (**d**).

PLGA-MCs were fabricated by proprietary technology utilizing dense gas (supercritical carbon dioxide) to provide a micrometric sized system for local sustained release of hTGF-β1 [32]. PLGA-MCs displayed a spherical shape with a mean size of 3 ± 0.9 µm (Figure 1a,b), but they were not suitable for conventional sterilization methods because of biopolymer oxidation or peptide payload degradation. To avoid these issues, a specific SEE operational protocol for sterile materials production was applied, which allowed for recovering the carriers in a sterile suspension [22]. 

The carriers were fabricated with an hTGF-β1 loading of 7 μg/g that provided a sustained release profile over 21 days into PBS at 37 °C (Figure 1c). A total load of 6.6 mg within each 3D collagen scaffold provided an actual hTGF-β1 release as illustrated in Figure 1d. The release at day one was 4.6 ng/mL followed by a mean concentration of about 7 ng/mL per day for the next six days. A concentration of 3.3 ng/mL/day across the following days was maintained, except at day 15, when a spike of 4.5 ng/mL resulted from PLGA initial depolymerization activity [45].

The release data profile was only reflective of the 3D scaffold when maintained in a sink condition provided in vitro by continuous stirring or perfusion. When same carriers were maintained in a static environment without medium replacement, the hTGF-β1 release was not observed or completely underestimated (data not shown). As a consequence, we supposed that the growth factor was not properly released within the 3D scaffold maintained in static conditions along culture period; conversely, the dynamic conditions by perfusion would better mimic the sink conditions required for proper drug release from PLGA carriers.

### 3.2. 3D Scaffold Environment Assembly and Characterization

**Figure 2:**
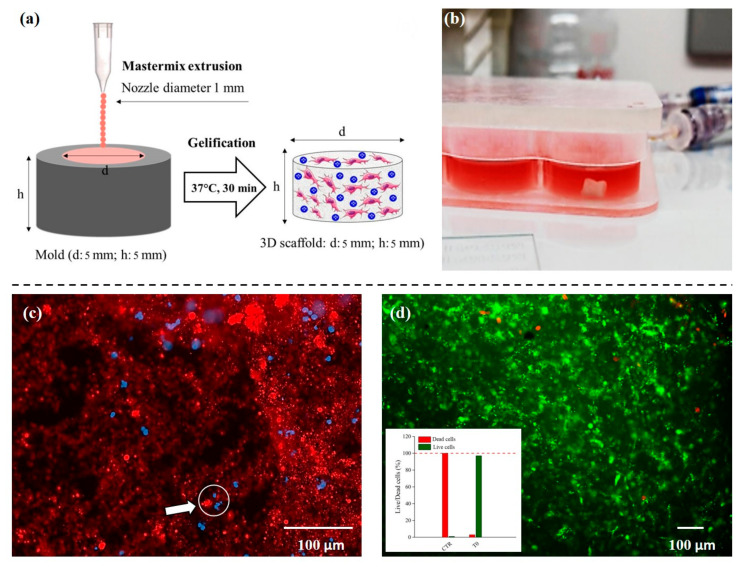
Production and characterization of the functionalized 3D scaffold. Schematic illustration of extrusion and gelification process of the collagen scaffold (**a**); image of the 3D system within the bioreactor after 16 days of culture (**b**). Confocal image of the scaffold integrated with hBM-MSCs (DAPI nuclei highlighted in blue) and PLGA-MCs loaded with Rhodamine B (highlighted in red) (**c**); live/dead signal evaluation of hBM-MSCs viability right after collagen extrusion and cross-linking (**d**).

A schematic representation of the 3D scaffold assembly protocol and resulting composite hydrogel is provided (Figure 2a,b). To better understand the impact on the scaffold architecture of coseeding with carriers and cells we performed additional characterization. PLGA-MCs loaded with rhodamine B (Rod-B, 8 mg/g) and hBM-MSCs were seeded in a scaffold assembly to observe carrier and cell dispersion. Dispersed elements, tracked by fluorescence and DAPI, used to highlight cell nuclei, demonstrated a homogeneous distribution within the scaffold (Figure 2c). A potential issue was that during mastermix extrusion, distribution of velocity and shear stress across the cross-section of the pipette nozzle would stress the cells loaded within the gel system, particularly at the wall areas [46]. We confirmed hBM-MSCs viability, following on from collagen cross-linkage, to be 97% (Figure 2d) which was reduced slightly to 90% following on from dynamic culture and further reduced to 80% in the static environment. 

**Figure 3:**
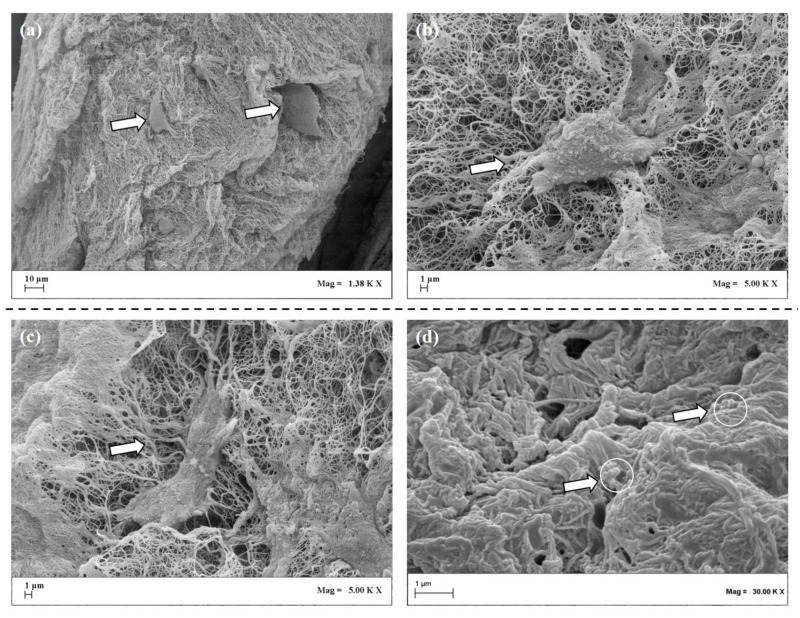
Freeze-dried scaffold observed by a Scanning Electron Microscope at different enlargements. The scaffold structure is formed by a network of fibers with inside cells distributed (**a**); collagen fibers are organized in randomly assembled web in which cells were clearly observed (arrowheads) (**b**,**c**); PLGA carriers are embedded within the collagen fibers (arrowheads with white circle) (**d**).

The collagen 3D scaffold was then fixed, dehydrated, and freeze-fractured before acquisition of FE-SEM images at different enlargements, where a collagen fiber network was observed (Figure 3a) with cells and PLGA carriers embedded within (Figure 3b–d).

### 3.3. Dynamic Culture by Perfusion Bioreactor

**Figure 4:**
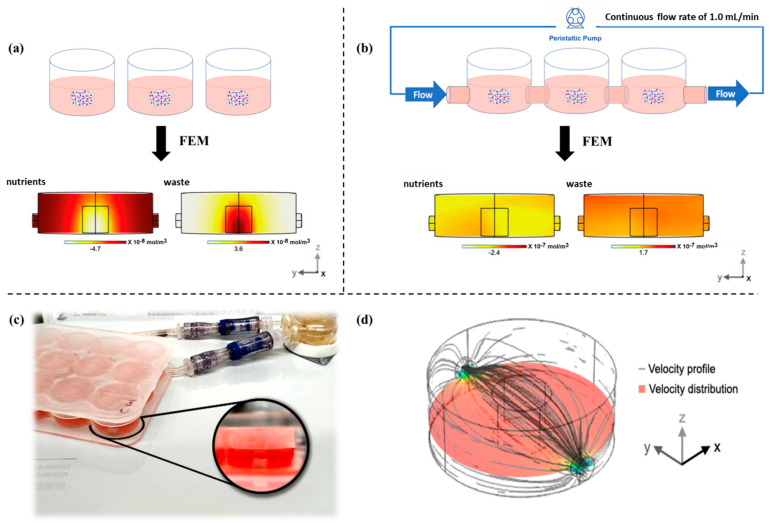
Perfusion culture bioreactor and FEM analysis of nutrients and waste mass exchange in static and dynamic conditions. Schematic illustration of the static (**a**) and dynamic (**b**) environment for the 3D scaffolds culture and related FEM analysis of nutrient consumption and waste production in both static vs. dynamic condition; molecule concentrations are represented as mean value and are expressed as mol/m^3^. Perfusion bioreactor images and 3D scaffold details (**c**) and FEM analysis of medium velocity profile (grey lines) and distribution (horizontal cross-section) within a single culture well (the perfusion flow is oriented along the y-direction) (**d**).

FEM analysis of nutrient concentration within a single culture well, in static vs. dynamic conditions, was performed (Figure 4a,b). Three-dimensional constructs were maintained in dynamic culture conditions with a1 mL/min continuous flow rate through each well (Figure 4b,c); in this condition, a laminar flow (grey lines) and a uniform velocity distribution (horizontal cross-section) were maintained within the culture wells (Figure 4d). In static conditions the in silico model demonstrated an uneven nutrient distribution inside wells, with the lowest observed concentration in the 3D construct (Figure 4a). Conversely, a more uniform nutrient distribution was maintained in the whole scaffold volume in dynamic conditions, as estimated by the order of magnitude of the nutrient concentration, 10^−8^ mol/m^3^ in static vs. 10^−7^ in the perfused environment (Figure 4b). The converse relationship was observed when performing waste product consideration (Figure 4a,b). These data reflect previous reports, where cells immobilized within a 3D system are under risk of reduced nutrient and oxygen exchange linked to impaired metabolic activity in static culture environments [21,47]. Moreover, overall mass exchange being favored by perfusion suggests an adequate environment to ensure appropriate sink conditions which enforce sustained release of growth factor from PLGA carriers dispersed within the 3D system [45]. The absence of proper mass exchange would negatively affect not only the nutrient and waste exchange but also the controlled release system behavior, preventing the achievement of adequate release profiles. The poor outcome of the static culture, where chondrogenic markers and cytokines were downregulated, may be linked to the suboptimal mass exchange that also prevented the hTGF-β1 release within the 3D system and, consequently, the proper cell stimulation.

**Figure 5:**
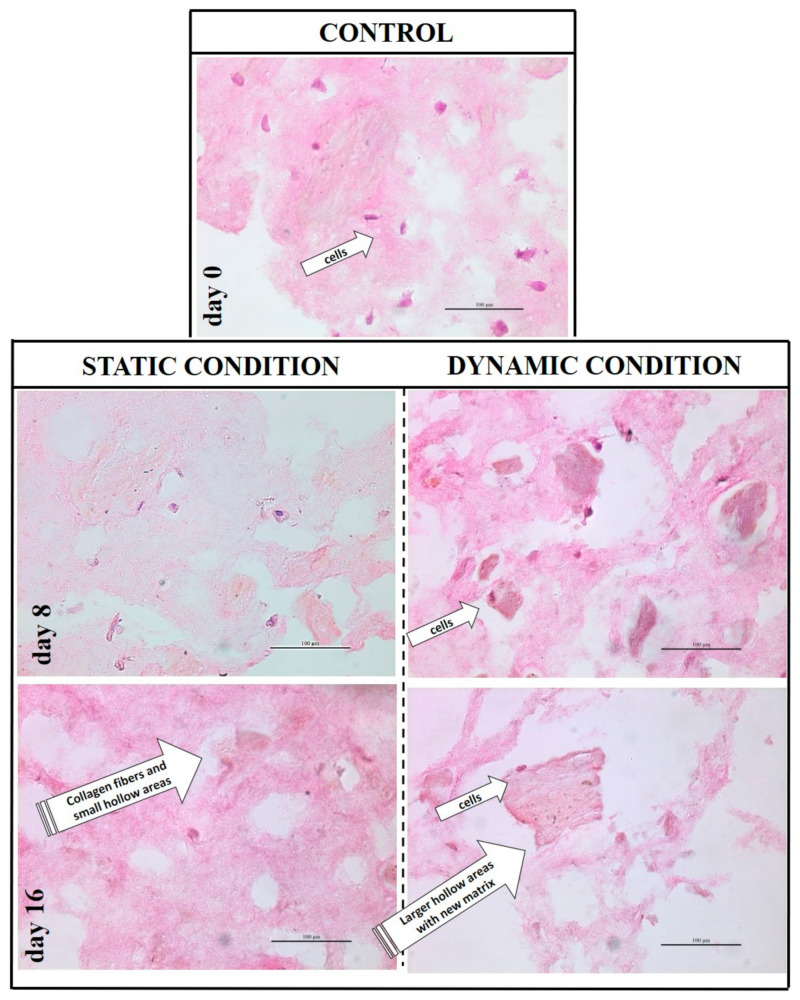
Hematoxylin & Eosin staining of 3D bioengineered system at different time-points in static and dynamic culture. An homogenous network of synthetic collagen matrix at day 0 was observed with cells immobilized within. The 3D collagen matrix maintained its integrity during the static culture; at day 16 small hollow areas were observed. On the contrary, same scaffolds cultured in dynamic condition appeared with an increased number of hollow areas, also larger. These areas appeared filled with a darker new matrix with cells clearly observed within (arrowheads). All images are obtained using an enlargements of 40×.

**Figure 6:**
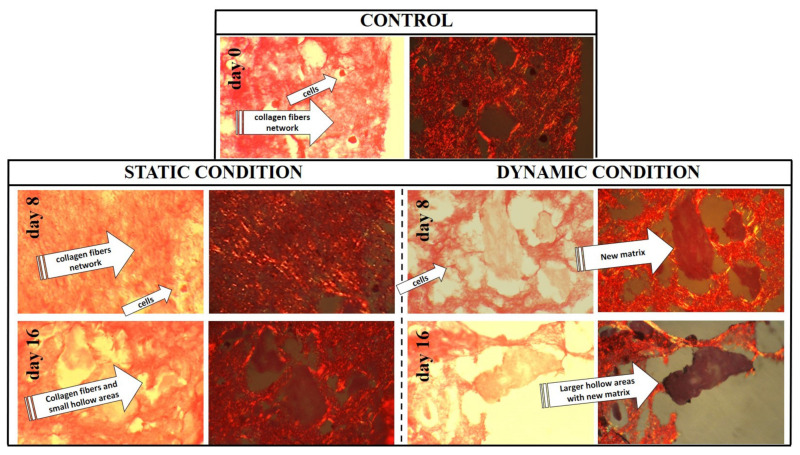
Polarized microscope images of 3D bioengineered scaffold stained by Sirius red. The collagen synthetic matrix is represented by network fibers stained in light red. This collagen matrix underwent a progressive degradation that was larger and more abundant in dynamic culture conditions. Furthermore, we observed new matrix deposition (darker red areas) within the hollow areas of the scaffold, but mainly at day 16 in the scaffold cultured in perfusion.). All images are obtained using an enlargements of 40×.

The collagen scaffold does not undergo any rearrangement or degradations during 16 days without cells cultured within, even if maintained in culture medium; furthermore, no changes in scaffold porosity, volume or color was monitored in absence of cell culture (data not shown). On the contrary, the same scaffold loaded with hBM-MSC underwent a change of its volume (reduced by 1/3) and color (whiter) after 16 days. Histological evaluation by H&E staining was applied to determine changes to the scaffold composition and structure during extended culture. At day zero, cells were homogeneously distributed within the hydrogel matrix and which itself appeared randomly assembled (Figure 5). The 3D collagen matrix maintained its integrity during the static culture and even at day 16 only small hollow areas were observed; conversely, the matrix surrounding the cells underwent a greater extent of remodeling in the dynamic regime, and distinct and novel regions deposition were also noted for cell-rich regions (see Figure 5, day 8 and 16 in dynamic conditions). This behavior was also confirmed by polarized microscope observation of the Sirius red-stained samples illustrated in Figure 6. The collagen synthetic fibers (in red) are evident along the culture evolution; however, they were progressively reshaped by the cells and several hollow areas appeared filled with darker red areas in where cells are clearly evident. These new matrix areas are more abundant in the dynamic culture and supposed to be new collagen, even if the fibers within appeared not yet organized.

### 3.4. 3D Microenvironment and hBM-MSC Chondrogenic Commitment: Gene Expression and Immunofluorescence Assay

**Figure 7:**
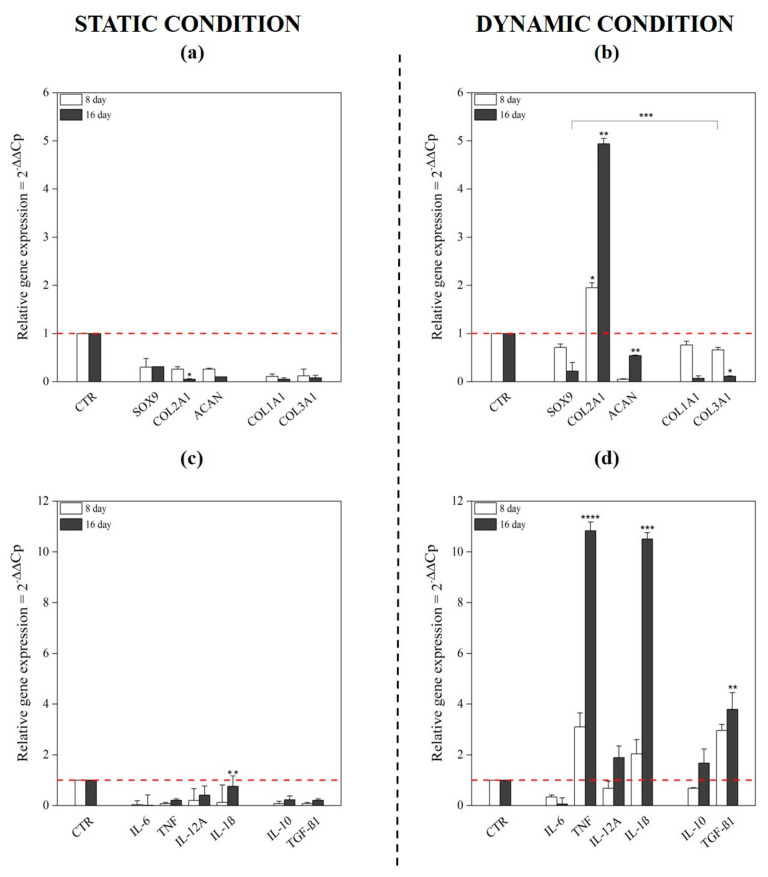
Gene expression profiling of chondrogenic markers and cytokines in static and dynamic culture conditions. The mRNA expression levels of positive and negative chondrogenic markers (COL1A1, COL2A1, COL3A1, SOX9 and ACAN) and cytokines (IL-6, TNF, IL-12A, IL-1β, IL-10 and TGF-β1) were monitored using a RT-qPCR; untreated cells were used as control. In static condition, all investigated genes were downregulated compared to the control (**a**,**c**). In contrast, COL2A1 mRNA levels showed, in dynamic culture, a significant upregulation (4.9-fold) at day 16 (**b**), as well as proinflammatory cytokines TNF and IL-1β (10-fold) and anti-inflammatory ones as IL-10 and TGF-β1 (2- and 3.8-fold changes) (**d**). * *p* < 0.05, ** *p* < 0.01, *** *p* < 0.001 and **** *p* < 0.0001 (two-way ANOVA, n = 3).

We next determined chondrogenic transcript expression within our scaffold and across the experimental timeline (Figure 7a,b). In the static condition, we observed marked downregulation of all transcripts tested (*SOX9*, *COL2A1*, *COL1A1*, *COL3A1*, and *ACAN*) at day 8 and further downregulation of all, except *SOX9*, at day 16. In contrast to the above, the dynamic condition stimulated *COL2A1* upregulation (2-fold, *p* < 0.05) at day 8 and further upregulation at day 16 (5-fold, *p* < 0.01). This supported the hypothesis of chondrogenic commitment by the cooperative effect of the growth factor and the dynamic culture. Conversely, *COL1A1* (0.07-fold) and *COL3A1* (0.11-fold, *p* < 0.05), fibrocartilage tissue markers [48], were both progressively downregulated across the time course. BM-MSC have an intrinsic tendency to differentiate towards an osteogenic lineage [21] and the marked downregulation of COL1A1 gene expression can be considered a further positive indication of commitment away from the default lineage.

*SOX9* upregulation was not observed in the 3D perfused culture. This is consistent with the previous reports describing *SOX9* expression as an early marker of chondrogenesis in 3D cultures, driving the regulation and activation of downstream targets including *COL2A1* [49,50]. Further, as an early marker of chondrogenesis, and consistent with the previous reports, its upregulation may have occurred prior to day 8 followed by a return to basal levels and below thereafter [51,52,53]. *ACAN* is reported to be a late marker [54] and while downregulated at day 8 we noted a subsequent significant upregulation from day 8 to day 16 in dynamic conditions.

**Figure 8:**
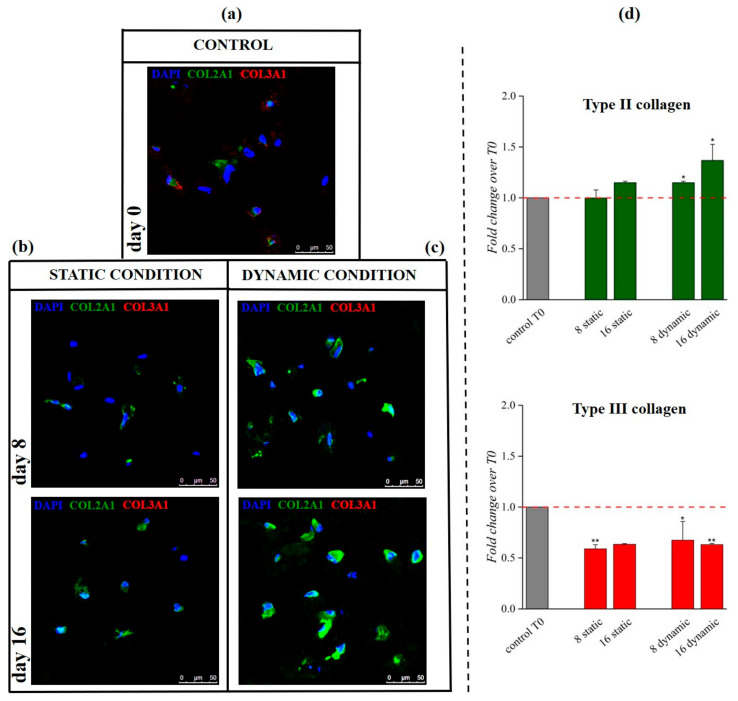
Immunofluorescence images and semiquantitative analysis of type II and III collagen signals in both static and dynamic 3D culture. IF assay at day 8 and 16 of static (**b**) and dynamic (**c**) culture showed an increase in type II collagen signal (green) especially in dynamic culture at day 16; type III collagen signal (red) was detected in the control sample at day 0 (**a**), but it decreased at the other time points. Q-IF confirmed this trend, indicating a statistically significant increase of type II collagen protein (stained in green) at day 16 in dynamic culture coupled with a significant reduction in type III collagen signal (stained in red) (**d**). Split channels and other IF data on type I collagen are reported in Supplementary Material. Scale bar: 50 µm. * *p* < 0.05, ** *p* < 0.01, (one-way ANOVA, n = 3).

Q-IF confirmed this trend, with increased type II collagen at day 16 in the dynamic culture (0.4-fold, *p* < 0.05) and reduced type I and III collagen throughout (*p* < 0.05) (Figure 8, Figures S1 and S2 in Supplementary Material); day 8 displayed higher levels of collagen II signal, which significantly increased at day 16 (0.4-fold, *p* < 0.05). The same investigation of collagen type I and III showed reduced signals at day 16 with respect to the control (see Figure 8d and Figure S2 in Supplementary Material) Changed expression in collagen II and collagen III and I were indicative of an early commitment towards the specific phenotype [34].

As a conceptual study to determine the suitability of scaffold to support chondrogenic differentiation and informative for future therapeutic applications we sought to determine the immunomodulatory reaction of cells to the synthetic environment by analysis of the hBM-MSCs cytokine gene expression. Proinflammatory cytokines, TNF and IL-1β displayed 10.8-fold and 10.5-fold upregulation (*p* < 0.0001) at day 16 in the dynamic culture (Figure 7d). This was coupled with the elevated expression of anti-inflammatory IL-10 (1.67-fold, *p* < 0.05) and TGF-β1 (3.80-fold, *p* < 0.01) at day 16 (Figure 7d). On the other hand, no cytokine expression was monitored in the static environment, suggesting that their overexpression was not induced by the 3D synthetic environment but more probably by cells’ commitment (Figure 7c). These data may also suggest that the environment itself does not stimulate cytokine upregulation but that the dynamic culture does in synergies of cell commitment versus a specific phenotype and opens perspectives for the potential use of these biomaterials (both PLGA carriers and collagen) as an injectable advanced therapeutical option for growth factor in situ delivery and local stem cell stimulation for cartilage tissue repair and healing.

## 4. Conclusions and Perspectives

The presented 3D environment, coupled to microcarrier-delivered hTGF-β1 release and perfusion culture-driven enhanced mass exchange, was effective in inducing chondrogenic commitment in hBM-MSCs. The versatility of the system enables future design for sustained release of multiple growth factors and/or immunomodulatory signals to further explore new solutions for in vitro cartilage repair and regeneration. Indeed, through the use of different MSCs loaded with distinct biological signals a complex spatial–temporal delivery could be achieved. Therefore, described 3D microenvironment therefore opens concrete perspectives for the development of 3D bioengineered models to understand the specific molecular and cellular composition of the damaged systems.

Looking to clinical application, collagen hydrogels enriched with PLGA MCs have a relatively low dynamic viscosity (10^4^ Pas) [43] and body temperature (37 °C) cross-linkage. This opens perspectives for their future use as intra-articular injectable advanced therapy for controlled-drug-delivery and/or factors for resident stem cell stimulation following in vivo administration.

## 5. Patents

The SEE technology for nanocarriers fabrication was described in the US Patent US/8628802 B2 Jan 2014. Inventors: Reverchon E., Della Porta G., “Continuous process for microspheres production by using expanded fluids. Applicant: University of Salerno. 

## Figures and Tables

**Table 1:** Mass transport parameters.

FEM Modeling Parameters	Values	Units
Glucose at time zero	0	mol/m^3^
Waste at time zero	0	mol/m^3^
Nutrient consumption	4.74·10^−12^	mol/s
Waste production	3.4·10^−12^	mol/s
D nutrients coefficient	3.9·10^−9^	m^2^/s
D waste coefficient	1.6·10^−9^	m^2^/s
κ collagen hydrogel	2·10^−9^	m^2^

## Data Availability

Not applicable.

## Supplementary Materials

The following are available online at https://www.mdpi.com/1999-4923/13/3/399/s1, Figure S1: Immunofluorescence images illustrating the expression of type II and type III collagen proteins by hBM-MSCs in both static and dynamic 3D culture, Figure S2: Immunofluorescence images illustrating the expression of type I collagen proteins by hBM-MSCs in both static and dynamic 3D culture at Day 16.

## Abbreviations

*h*TGF-β1	Human transforming growth factor β1
PLGA-MCs	Poly-lactic-co-glycolic acid microcarriers
hBM-MSCs	Human bone marrow mesenchymal stem cells
hASC	Human adipose stem cells
COL1A1	Type I collagen
COL2A1	Type II collagen
COL3A1	Type III collagen
SOX9	SRY-Box transcription factor 9
ACAN	Aggrecan
RT-qPCR	Reverse transcription quantitative polymerase chain reaction
q-IF	Semiquantitative immunofluorescence
