# Supplementary material for: Chondrogenic Commitment of Human Bone Marrow Mesenchymal Stem Cells in a Perfused Collagen Hydrogel Functionalized with hTGF-β1-Releasing PLGA Microcarrier
Source: Pharmaceutics. 2021 Mar 17;13(3):399. doi: 10.3390/pharmaceutics13030399 (PMC8002618; doi:10.3390/pharmaceutics13030399)
Supplement: Supplementary file 1 [file pharmaceutics-13-00399-s001.pdf]

pharmaceutics

MDPI

Supplement Materials: Chondrogenic Commitment of Human Bone Marrow Mesenchymal Stem Cells in a Perfused, hTGF-β1-Releasing PLGA Microcarrier Seeded, Collagen Hydrogel

Erwin Pavel Lamparelli, Joseph Lovecchio, Maria Camilla Ciardulli, Valentina Giudice, Tina P. Dale, Carmine Selleri, Nicholas Forsyth, Emanuele Giordano, Nicola Maffulli and Giovanna Della Porta

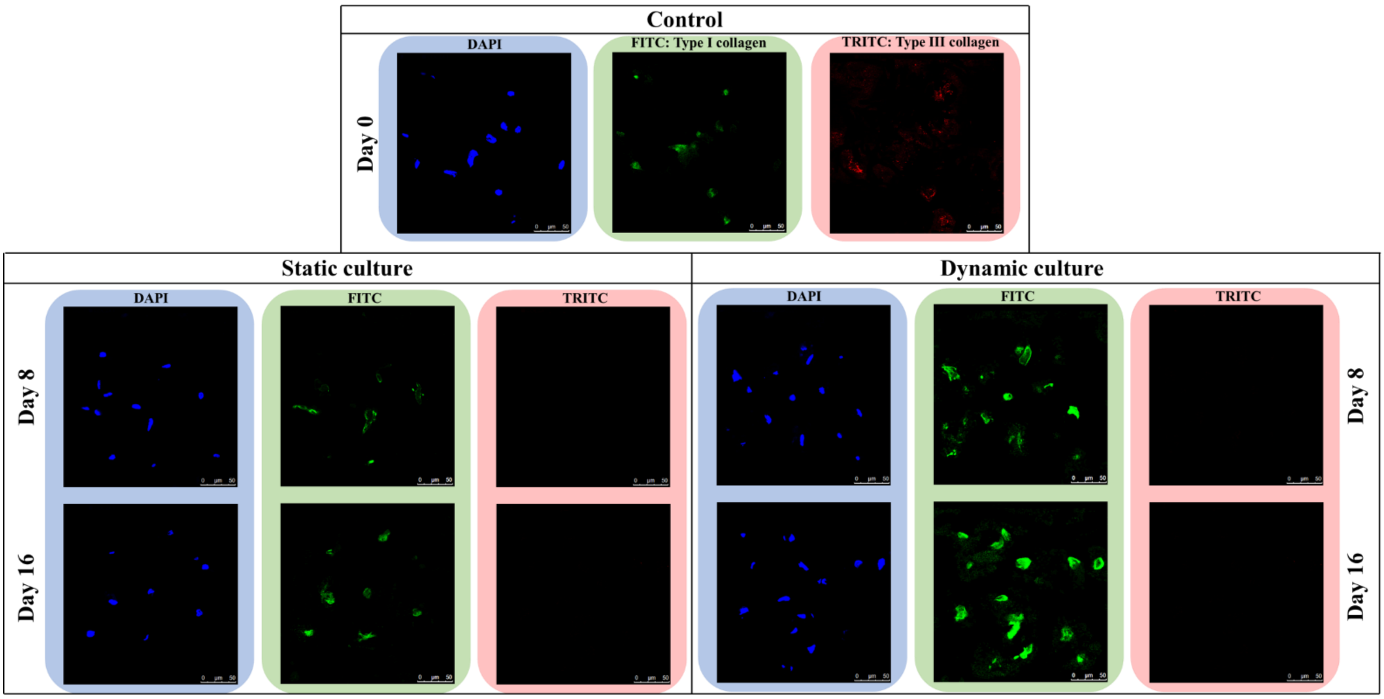

Figure S1. Immunofluorescence images illustrating the expression of type II and type III collagen proteins by hBM-MSCs in both static and dynamic 3D culture. The panel shows the split color channels for type II Collagen (green staining) and type III Collagen (red staining) proteins expressed by hBM-MSCs at Day 8 and 16 of culture. The Collagen II protein was better stained in the samples slices obtained from 3D culture in dynamic conditions, confirming the qRT-PCR data. Collagen Type III was down regulated along the 3D culture. Scale bar: 50 μm.

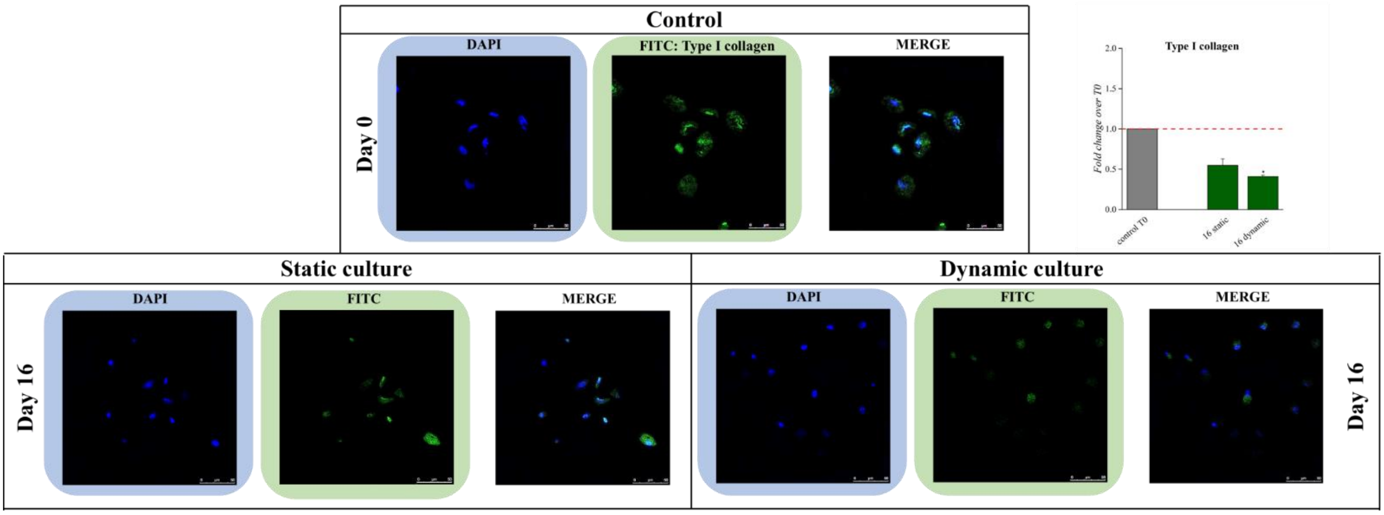

Figure S2. Immunofluorescence images illustrating the expression of type I collagen proteins by hBM-MSCs in both static and dynamic 3D culture at Day 16. The panel shows type I Collagen (green staining) proteins expressed by hBM-MSCs at Day 8 and 16 of culture; it appeared down regulated along the 3D culture, in both static and dynamic conditions, confirming the qRT-PCR data. Quantitative analysis of the signal performed by Image J software indicated that the protein was downregulated at Day 16 better in dynamic culture conditions. Scale bar: 50 μm.
